# Proximal Interphalangeal Joint Surface Replacement Arthroplasty Using the WALANT Technique by Palmar Approach: A Case Report

**DOI:** 10.1155/cro/5445262

**Published:** 2026-09-30

**Authors:** Fidel Ernesto Cayon Cayon, Gabriel Fernando Alegría Velasco, Paul Santiago Vaca Perez, Carlos Patricio Peñaherrera Carrillo, Francisco Endara Urresta, Alejandro Xavier Barros Castro, Diego Patricio Michilena Chávez

**Affiliations:** ^1^ Hospital Metropolitano de Quito Quito Ecuador; ^2^ Metropolitan Hospital International University of Ecuador Quito Ecuador; ^3^ Vozandes Hospital Quito Ecuador

**Keywords:** case report, proximal interphalangeal arthroplasty, proximal interphalangeal osteoarthritis, surgical technique, WALANT technique

## Abstract

Osteoarthritis of the proximal interphalangeal joint (PIPJ) is a common condition characterized by pain, weakness, stiffness, malalignment, and loss of movement in the affected hand. Conservative treatments such as medication, physical therapy, and splinting, as well as surgical procedures like synovectomy and debridement, may occasionally help; however, arthrodesis and PIPJ arthroplasty are the definitive surgical options. The goal of replacement arthroplasty is to relieve pain, restore movement, and maintain the stability of the affected joint. Designing an effective replacement arthroplasty requires a deep understanding of current biomaterials and an appreciation for the complex biomechanics of the PIP joint. The use of local anesthetic (wide‐awake local anesthesia no tourniquet [WALANT]) offers advantages such as the absence of discomfort from a tourniquet, reduced personnel and sedation costs, faster recovery, and intraoperative assessment of motor function. Our objective is to report a case in which a PIPJ arthroplasty was successfully performed using the WALANT technique.

## 1. Introduction

Arthritis of the proximal interphalangeal joint (PIPJ) is a common source of pain, stiffness, deformity, and functional impairment of the hand. Osteoarthritis, posttraumatic degeneration, and inflammatory arthropathies may all affect this joint, although their clinical presentation and treatment considerations may differ. When conservative management fails and symptoms remain disabling, surgical treatment may include arthrodesis or joint replacement, with the choice depending on factors such as the affected digit, joint stability, deformity, bone quality, and the patient′s functional requirements [1, 2].

The PIPJ has a complex anatomical configuration that allows flexion and extension while also accommodating limited multiplanar motion. Its stability depends on the relationship between the articular surfaces, collateral ligaments, volar plate, and surrounding soft tissues. The geometry of the proximal and middle phalanges is not completely symmetrical, and the morphology of the joint varies between the different fingers. These characteristics contribute to the functional behavior of the joint and represent an important consideration when planning reconstructive procedures. [3–6]

PIP joint arthroplasty has evolved considerably since the introduction of early implant designs. Silicone implants, particularly the Swanson prosthesis, have been widely used; however, their limitations have encouraged the development of alternative implant concepts intended to reproduce joint anatomy while preserving motion. Surface replacement systems have subsequently been introduced as an alternative to arthrodesis in appropriately selected patients, particularly when preservation of PIP joint motion is considered functionally desirable. Successful implantation depends on adequate bone stock, joint stability, appropriate soft‐tissue balance, and careful patient selection [2, 7–9].

The biomechanical characteristics of the PIPJ present particular challenges for implant design. Unlike larger joints, the small dimensions of the PIPJ provide limited tolerance for errors in implant positioning, while the surrounding ligamentous and tendon structures have an important role in maintaining alignment and motion. Consequently, contemporary PIP arthroplasty has focused not only on implant materials but also on implant geometry and the preservation of the soft‐tissue structures that contribute to joint stability [4, 7].

Wide‐awake local anesthesia no tourniquet (WALANT) has become increasingly used in hand surgery because it allows selected procedures to be performed under local anesthesia without a tourniquet. The technique may facilitate outpatient treatment, avoid tourniquet‐related discomfort, reduce the need for sedation, and permit assessment of active movement during surgery when appropriate. WALANT has been described for a broad range of hand and wrist procedures, including increasingly complex reconstructive interventions [10].

In this report, we describe a case of PIPJ surface replacement arthroplasty performed using WALANT through a volar Z‐shaped approach. The purpose of this report is to describe the surgical technique and the clinical outcome observed during follow‐up.

## 2. Case Presentation

A 36‐year‐old male patient with no significant personal medical history reports that 9 months prior to his evaluation, he sustained trauma to the fourth finger of his right hand after falling from a motorcycle, although he is unaware of the exact mechanism of the injury. Following this incident, he began to experience constant pain localized in the PIPJ of the ring finger, which increases with drops in ambient temperature, along with deformity and difficulty achieving full flexion and extension of the described joint, progressively worsening over time.

Physical examination reveals tenderness on palpation in both the dorsal and volar regions over the PIPJ of the right fourth finger, with negative ulnar and radial stress tests. Range of motion is limited, with flexion at 70° and extension at −2°, exhibiting a varus deformity (Figure 1).

**Figure 1:**
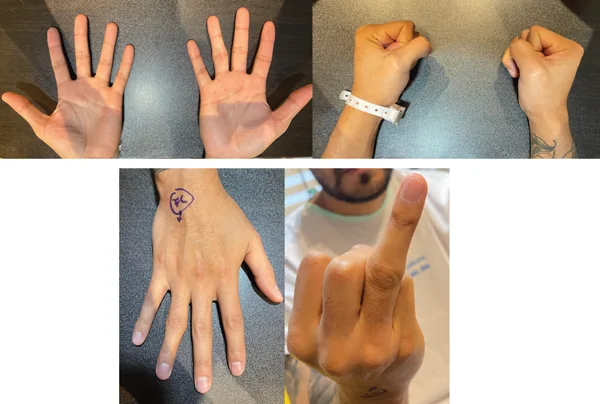
Clinical images.

A radiographic study is requested in two projections (anteroposterior and lateral), which shows an increase in soft tissue volume with ulnar deviation of the middle phalanx of the fourth finger and loss of joint space at the proximal interphalangeal level, along with a subcortical cyst at the base of the middle phalanx (Figure 2).

**Figure 2:**
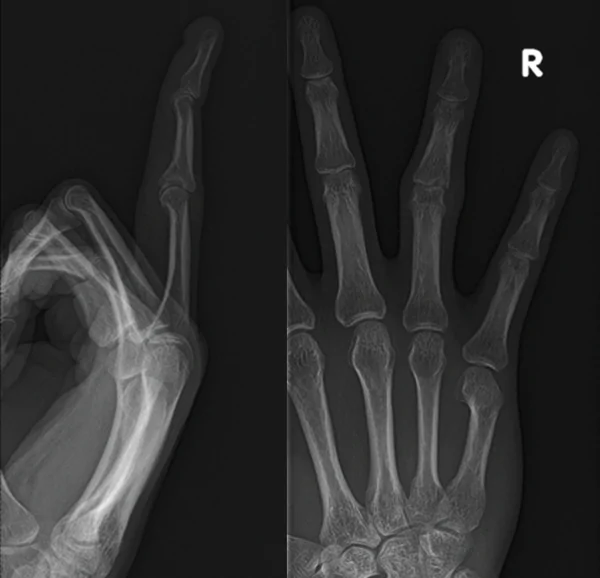
Lateral and A‐P radiography of the fourth finger of the right hand.

An evaluation is complemented with a simple MRI of the hand, which shows advanced posttraumatic osteoarthritis in the PIPJ of the fourth finger, with depression of the articular surface at the base of the middle phalanx and avulsion of the volar plate, indicating the need for surgical intervention (Figure 3).

**Figure 3:**
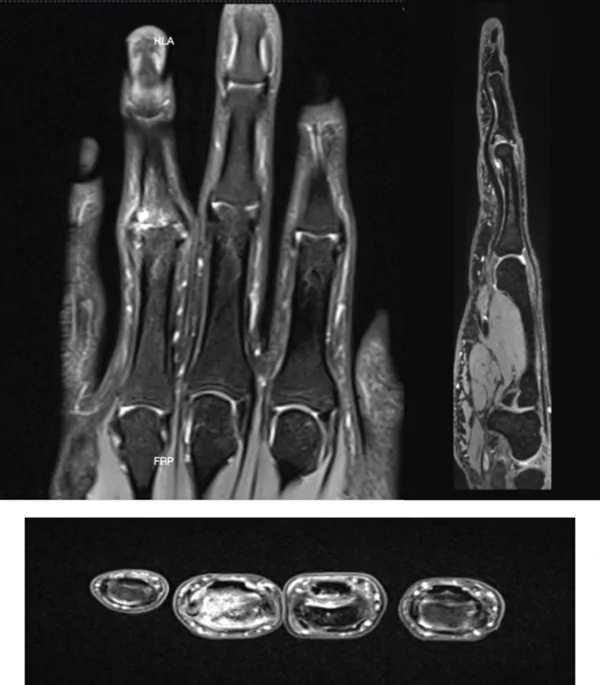
Simple MRI of the right hand.

The indications for prosthesis implantation include degenerative and posttraumatic osteoarthritis of the PIP joint, and occasionally inflammatory arthritis [9, 11]. Therefore, a surface replacement arthroplasty of the PIPJ of the right hand′s ring finger was performed. The procedure began with the administration of local anesthetic using the WALANT technique (1% lidocaine with 1:100,000 epinephrine was used, infiltrating approximately 10 mL into and around the base of the joint to block the digital nerves via circumferential subcutaneous infiltration to achieve adequate anesthesia and hemostasis. The infiltration was performed 30 min before the surgical incision to allow for maximum vasoconstrictor effect of the epinephrine. The total dose was kept within the safe limit of 7 mg/kg of lidocaine.), which is currently an attractive option as it eliminates the dependency on major operating rooms or hospital procedures, saves costs, and the associated complications are minimal, including vasovagal syncope and cardiac arrhythmia due to inadvertent intravascular injection of epinephrine. Access was achieved through a Z‐incision over the volar area of the joint, allowing access to the volar plate and the articular surface (Figure 4).

**Figure 4:**
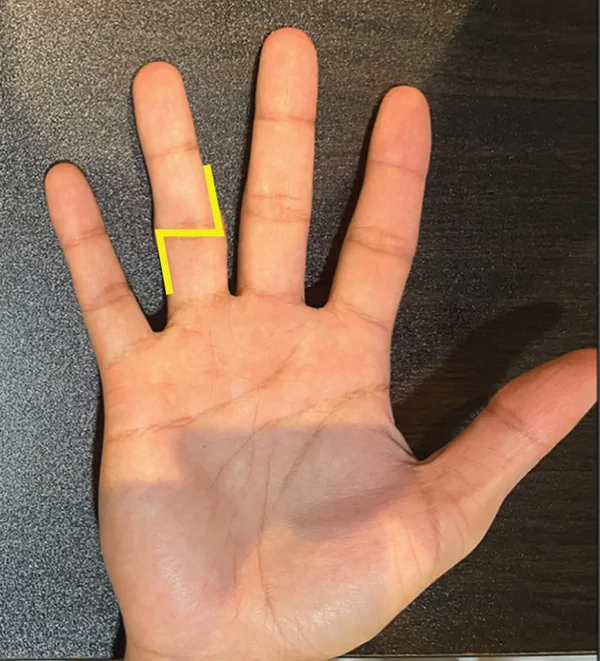
Z‐approach volar.

The incision is extended to the flexor sheath, and the neurovascular bundles on each side are visualized and mobilized. This is particularly important to avoid excessive stretching of the bundles. The flexor sheath is excised between the A2 and A4 pulleys, and the flexor tendons are retracted (Figure 5). Alternatively, the FDP can be retracted, and the FDS can be splinted in line with its fibers to visualize the volar plate. The accessory collateral ligaments are then incised along the volar plate, which is released from the base of the middle phalanx. This now allows access to the articular surface.

**Figure 5:**
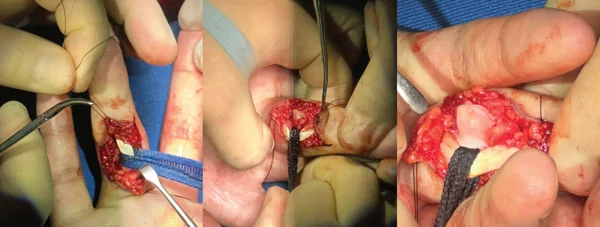
Retraction of the flexor sheath and exposure of the PIPJ.

Osteophytes were removed, and joint separation was performed for the bone cuts, which were made perpendicular to the axis using an oscillating saw on both phalanges (Figure 6). Reaming was performed at the level of the middle phalanx to prepare the canal. Trial implants were tested for stability and their placement was verified with a fluoroscope; subsequently, the definitive implants were placed, and a dynamic check was performed (Figure 7).

**Figure 6:**
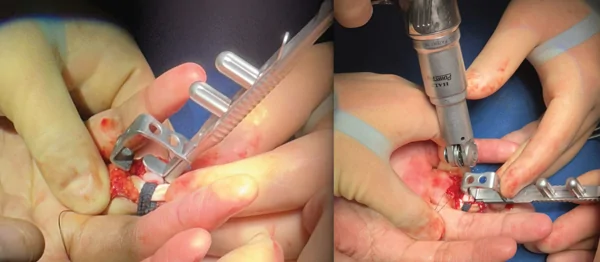
Bone cuts with oscillating saw.

**Figure 7:**
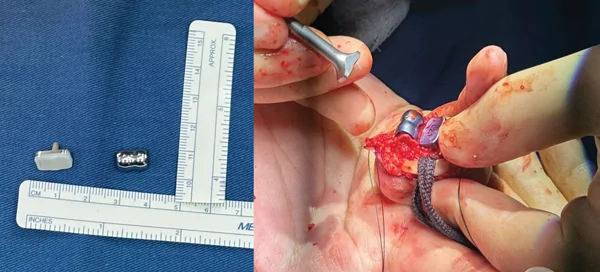
Placement of definitive CapFlex implant.

During closure, the volar plate must be repaired; however, the collateral ligaments and the flexor sheath can be left unrepaired without risking significant instability. In our case, we repaired the volar plate with the respective anchoring of the accessory collateral ligaments, closed the wound in layers, and applied sterile dressings and a bulky bandage with Coban wrap. The procedure was completed without immediate complications, allowing for early mobilization of the joint. Postoperative dynamic radiographs were performed to rule out malalignment, with all results being negative (Figure 8).

**Figure 8:**
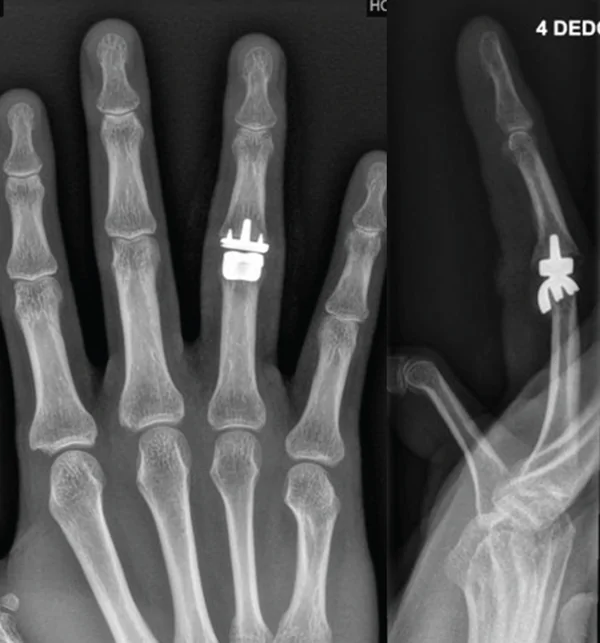
Postoperative images.

During the 12‐month clinical follow‐up after PIPJ articular surface replacement performed using the WALANT technique via a volar Z‐approach, the patient experienced no pain (VAS 0/10) or procedure‐related complications. The final range of motion was −20° of extension and 0°–90° of flexion of the PIPJ. Furthermore, the patient reported high satisfaction with the functional outcome and the improvement in activities of daily living (Figure 9).

**Figure 9:**
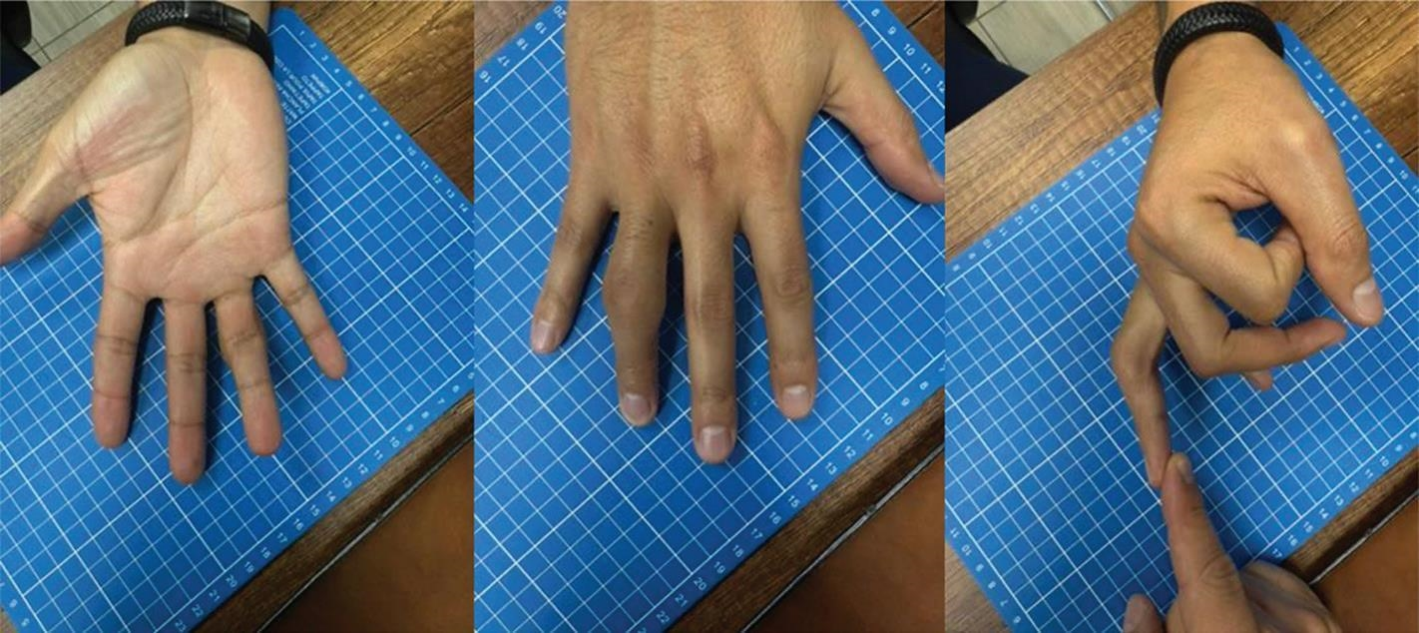
1‐year follow‐up images.

## 3. Discussion

The optimal surgical treatment for symptomatic PIPJ arthritis remains dependent on the characteristics of the individual patient and the affected joint. Arthrodesis provides reliable pain relief and stability but eliminates motion at the involved joint. In contrast, arthroplasty is intended to relieve pain while preserving functional movement. However, implant‐related complications such as loosening, fracture, instability, and dislocation remain important considerations when selecting joint replacement [2, 9, 12–15].

The development of PIPJ arthroplasty has involved multiple implant designs and surgical strategies. Early prosthetic concepts were followed by silicone implants and, subsequently, surface replacement systems designed to reproduce the native joint more closely. Despite these advances, outcomes remain influenced by implant characteristics, bone stock, joint alignment, ligamentous stability, and the integrity of the surrounding soft tissues [9, 12–15].

The surgical approach is another relevant aspect of PIPJ arthroplasty. Dorsal, volar, and lateral approaches have been described, each providing different exposure while requiring different considerations regarding the extensor mechanism, volar structures, collateral ligaments, and postoperative rehabilitation. The dorsal approach provides direct access to the joint but involves manipulation of the extensor apparatus. Volar approaches preserve the extensor mechanism but require careful management of the neurovascular bundles, flexor tendons, and volar plate. Current evidence has not established a universally superior approach for PIP arthroplasty [16–18].

In a retrospective comparison of volar and dorsal approaches, Tranchida et. al. found no statistically significant difference between the groups in postoperative range of motion, overall complication rates, or revision surgery, although the change in range of motion differed between approaches. These findings support the importance of considering the specific clinical circumstances and surgeon experience when selecting the surgical approach [17].

In our case, a volar Z‐shaped incision was used to obtain access to the PIPJ while permitting direct visualization of the volar plate and articular surfaces. The neurovascular bundles were identified and protected, and the flexor sheath was managed to allow exposure of the joint. The volar plate and accessory collateral ligament structures were subsequently repaired during closure. The approach allowed early postoperative mobilization, which was considered desirable in view of the objective of preserving joint motion.

WALANT was used as the anesthetic technique in this procedure. The use of local anesthesia without a tourniquet may provide several practical advantages in selected hand procedures, including avoidance of tourniquet discomfort, reduced requirements for sedation, and the possibility of performing surgery in an ambulatory setting. In addition, the technique permits active assessment of finger movement during surgery when clinically appropriate [10].

At 12 months, the patient reported no pain and no procedure‐related complications. The PIPJ demonstrated a range of motion from −20° of extension to 90° of flexion, and the patient reported a high level of satisfaction with the functional result and improvement in activities of daily living. These findings represent the outcome of a single case and therefore cannot be used to establish the effectiveness or superiority of the described technique.

An additional aspect of this case is the use of a volar Z‐shaped approach for PIPJ surface replacement arthroplasty. Based on our review of the available literature, we did not identify previous publications specifically describing this incision for PIPJ surface replacement arthroplasty. The present observation should therefore be considered a technical description rather than evidence of superiority over established approaches. Further comparative studies are required to determine whether this approach offers meaningful advantages regarding exposure, soft‐tissue preservation, postoperative motion, complications, or functional outcomes.

## 4. Conclusions

This article is one of the first studies reported regarding the use of the WALANT technique in PIPJ arthroplasty. However, we still need more prospective studies to help identify potential complications or benefits of this technique in both the short and long term.

Furthermore, based on our review of the available literature, no previous publications specifically describing a volar Z‐shaped approach for PIPJ surface replacement arthroplasty were identified. This approach could potentially reduce the risks observed with other volar approaches. However, further prospective and comparative studies are needed to determine its clinical significance and to validate its potential advantages.

The effective use of replacement arthroplasty requires a deep understanding of current biomaterials and an appreciation of the complex biomechanics of the PIP joint, tailored to each patient.

## Funding

No funding was received for this manuscript.

## Disclosure

The authors declare that no patient data appear in this article.

## Ethics Statement

The authors declare that the procedures conformed to the ethical standards of the responsible human experimentation committee and in accordance with the World Medical Association and the Declaration of Helsinki.

## Consent

Informed consent was obtained from the patients who participated in the study.

## Conflicts of Interest

The authors declare no conflicts of interest.

## Data Availability

The data that support the findings of this study are available from the corresponding author upon reasonable request.
